# Nanoemulsion of myricetin enhances its anti-tumor activity in nude mice of triple-negative breast cancer xenografts

**DOI:** 10.3389/fonc.2025.1563076

**Published:** 2025-06-09

**Authors:** Preeti Sharma, Yogesh Rai, Mohammad Ahmed Khan, Anant Narayan Bhatt, Abul Kalam Najmi, Shubhra Chaturvedi, Mohd. Akhtar, Anil Kumar Mishra

**Affiliations:** ^1^ Department of Pharmacology, School of Pharmaceutical Education and Research, Jamia Hamdard University, Delhi, India; ^2^ Division-Radiological Imaging and Neurocognition, Radiopharmaceutical Chemistry Laboratory, Institute of Nuclear Medicine and Allied Sciences (INMAS), Defence Research and Development Organisation (DRDO), Timarpur, Delhi, India; ^3^ Radiation and Cell Signaling Research, Institute of Nuclear Medicine and Allied Sciences (INMAS), Defence Research and Development Organisation (DRDO), Timarpur, Delhi, India; ^4^ Department of Chemistry, Indian Institute of Technology (IIT), Roorkee, Roorkee-Uttarakhand, India

**Keywords:** TNBC tumor, myricetin, nanoemulsion, PI3K/Akt/mTOR signaling, VEGFR2

## Abstract

**Purpose:**

Myricetin, a naturally occurring flavonoid exhibits good anti-cancer properties. However, its practical application is limited due to poor aqueous solubility and low bioavailability. To overcome these challenges, a nanoemulsion-based formulation of myricetin was developed and its anti-tumor efficacy was compared with Myricetin alone in TNBC xenografts.

**Methods:**

Athymic nude mice were randomly divided into three groups (n=8) of control, Myricetin (50mg/kg), Myr-NE (25mg/kg), and subcutaneously implanted with MDA-MB-231 cells. After the 7-day treatment regimen, tumor volume was measured for up to 21 days, followed by mechanistic investigation, including tumor histology and immunoblotting. Tumor migration, invasion, cell proliferation kinetics, clonogenic, oxidative stress, and nuclear fragmentation studies were performed in tumor-derived cells. ANOVA test was further performed for statistical analysis to assess the significance between the experimental groups.

**Results:**

Myr-NE treatment substantially reduced tumor progression compared to Myricetin alone in TNBC xenografts. The invasion, proliferation, and clonogenicity of Myr-NE tumor-derived cells were significantly reduced compared to Myricetin. The mechanistic investigation revealed that Myr-NE treatment effectively inhibits the PI3K/AKT/mTOR signaling and VEGFR2, accompanied by a significant reduction in the level of tumorigenic factors, including HIF-1α, Ki67, and MMP9 proteins compared to Myricetin. Myr-NE treatment also showed increased oxidative stress and DNA damage, resulting in enhanced tumor cell death compared to Myricetin alone.

**Conclusion:**

Similar to our earlier observation in *in-vitro* TNBC model, findings in the present study highlights that nanoemulsion of myricetin potentiates its anti-tumor activity in TNBC xenografts and provide a promising drug delivery strategy for better clinical outcomes.

## 1 Introduction

Triple-negative breast cancer (TNBC) is characterized by the lack of estrogen receptors (ER), progesterone receptors (PR), and human epidermal growth factor receptors (HER2) (1). Due to the absence of ER, PR, and HER2 receptors, TNBC does not exhibit a favorable response to hormone therapy or HER2-targeted therapy, both of which are routinely employed treatments for other forms of breast cancers (2). Mounting evidence suggests that phosphoinositide 3 kinase (PI3K)/Akt/mammalian target of rapamycin (mTOR) signaling pathway (PAM) is commonly altered in TNBC that drives tumor progression and therapy resistance (3–5). The primary challenge with the current treatment regimen is the development of therapy resistance and the occurrence of adverse side effects, which has redirected the focus towards medicines with lower toxicity and higher efficacy. To enhance the therapeutic benefits, it is crucial to identify PAM inhibitors that selectively target tumor signaling with the lowest adverse impact. Due to the significant enhancement in treatment effectiveness and the substantial decrease in adverse impacts, the utilization of natural constituents for drug development is crucial in the pharmaceutical industry (6). A naturally occurring flavonoid like Myricetin found in plants is gaining recognition for its anti-cancer potential through the selective inhibition of the PAM pathway (7–9). However, the practical application of flavonoids is limited due to their poor aqueous solubility and low bioavailability (10). In this context, nanoemulsions are reported to exhibit potential solutions with safe-grade excipients, primarily employed as carriers for drugs with poor aqueous solubility, low bioavailability, premature release, compromised targeted orientation, and loading capacity (10–12). Therefore, a nanoemulsion-based drug formulation was developed to identify the precise effectiveness of flavonoid Myricetin against TNBC in both *in-vitro* and *in-vivo* model systems. Our recent *in-vitro* study showed that nanoemulsion significantly enhances solubility, stability, *in-vitro* drug release, and intracellular internalization of Myricetin, eventually potentiating its anti-cancer activity in the TNBC cell line (13). Our study demonstrates that nanoemulsion augmented the efficacy of Myricetin by effectively inhibiting the PAM pathway, ultimately leading to enhanced cell death in TNBC cells (13). Based on this compelling evidence, the present study aimed to perform a pre-clinical investigation on the effect of Myrcietin and Myr-NE treatment in TNBC xenografts.

Our results demonstrate that the activation of PAM signaling and tumorigenesis-associated proteins are efficiently downregulated by Myr-NE treatment, leading to a significant decrease in tumor growth compared to Myricetin alone in TNBC xenografts. Moreover, tumor-derived cells showed a notable reduction in clonogenic survival, migration, and invasion potential, increased oxidative stress, and DNA damage, contributing to enhanced cell death in Myr-NE tumors compared to Myricetin tumor xenografts. Results obtained from the study support and validate our hypothesis that nanoemulsion has the potential to overcome the limitations associated with flavonoids and can be used as an efficient drug delivery strategy for TNBC treatment.

## 2 Materials and methods

### 2.1 Reagents and chemicals

The cell growth medium Dulbecco’s Minimum Essential Medium (DMEM) high glucose (Cat#D1152), antibiotics, penicillin G, streptomycin, and antifungal nystatin, were acquired from Sigma Chemicals Co. (St Louis, U.S.A.). Fetal bovine serum (FBS; Cat#16000-044) was purchased from Invitrogen. Primary antibodies PI3K (Cat#4249S), p-PI3K (Cat#4228), Pan AKT (Cat#C67E7), p-AKT (Cat#S473), m-TOR (Cat#2972S), p-m-TOR (Cat#2971S), HIF-1α (Cat#3716S), VEGFR2 (Cat#2479S), MMP-9 (Cat#13667S), Catalase (Cat#12980S), Bax (Cat#2772S), Bcl-2 (Cat#MA5-11757), RAD-51 (Cat#8875S), and Ku70 (Cat#4103S), were procured from Cell Signaling Technology (Danvers, MA, USA). Ki67 (E-AB-31869) was purchased from Elabsciences, whereas β-Actin (612657) was obtained from BD Biosciences (CA). Horseradish Peroxidase (HRP) conjugated secondary antibodies were obtained from Thermo Fisher Scientific (USA). Myricetin (Item No. 10012600) was obtained from Cayman Chemical Co. (USA).

### 2.2 Preparation and characterization of myricetin-nanoemulsion

The Myricetin-loaded nanoemulsion (Myr-NE) was prepared using our previously described spontaneous emulsification method (13). Briefly, Myr-NE was formulated using oil, surfactant, and cosurfactant, namely Capryol 90, Tween 20, and Transcutol HP, respectively. Further characterization study was carried out using dynamic light scattering (DLS) technique to estimate the size, zeta potential, and Polydispersity index (PDI) of the optimized nanoemulsion. Additionally, transmission electron microscopy (TEM) was performed for morphological assessment, and the thermodynamic stability was confirmed by the Heating-cooling cycle, Freeze-thaw cycle, and centrifugation test (13). The drug encapsulation was estimated, and finally, the *in-vitro* release property of Myr-NE was compared with Myricetin alone. The characterization study showed that the developed Myr-NE has a spherical shape and was stable. It exhibits particle size (89.76 nm), PDI (0.105), zeta potential (-6.33 mv), and drug loading content of 98%, which eventually resulted in increased *in-vitro* drug release and enhanced intracellular internalization as compared to Myricetin alone (13).

### 2.3 Drug treatment procedure

The dose of Myricetin (50 mg/kg) was opted for the TNBC xenograft treatment based on the earlier reported *in-vivo* studies (14–17). However, the dose of Myr-NE was kept at 25mg/kg body weight. Here, the rationale behind selecting this half dose rather than Myricetin is based on our earlier *in-vitro* study in TNBC (MDA-MB-231) cells. A substantial increase in the Myr-NE-induced anti-cancer efficacy was observed at more than 2-fold reduced IC50, compared to Myricetin alone (13). This observation led us to the comparative efficacy evaluation at a reduced dose of Myr-NE compared to Myricetin alone. Myricetin and Myr-NE administration was started on the 5^th^ day of tumor implantation (daily up to 7 days) via intraperitoneal (i.p.) route when the tumor palpability was observed in tumor xenografts. The control group was treated with the blank nanoemulsion vehicle identical in composition to the Myr-NE formulation but without Myricetin.

### 2.4 Source of tumor cell line

The MDA-MB-231 cell line was obtained from the National Centre for Cell Sciences (NCCS) in Pune, India. Cells were grown in DMEM high glucose supplemented with 10% FBS, 1% penicillin-streptomycin, and incubated at 37°C in a humidified incubator containing 5% CO_2_. Cell cultures were maintained using routine passaging (every 2-3 days) in 60 mm tissue culture dishes (BD Falcon, U.S.A.).

### 2.5 Ethics statement

Animals (24) used for the tumor study were obtained from the in-house animal facility of the Institute of Nuclear Medicine and Allied Sciences. All animals were cared for and fed in accordance with the recognized norms for the maintenance and use of laboratory animals throughout the research period, as outlined elsewhere (18). Humane endpoints were defined as tumor volumes exceeding 1,100 mm³, tumor ulceration, necrosis, infection, significant distress, weight loss of ≥20%, or impaired mobility. Mice were euthanized using carbon dioxide (CO₂) inhalation, administered at a controlled rate of 30–70% of chamber volume per minute to minimize distress. Death was verified by the absence of respiratory movement, heartbeat, and corneal reflex, followed by cervical dislocation as a secondary confirmation method. The animal experiment protocols were reviewed and approved by the Institutional Ethical Committee (IEC) at the Institute of Nuclear Medicine and Allied Sciences, referenced as INM/IAEC/2022/18 and INM/IAEC/2019/01.

### 2.6 Tumor study

The study on tumor xenografts utilized female athymic nude (BALB/c) mice that were six to eight weeks old, weighing 20-25g. The mice were kept in an environment with optimal temperature (23-25°C) and relative humidity (55%). Prior to the commencement of the study, mice were acclimated for one week and randomly divided into three distinct groups, namely Control, Myricetin, and Myr-NE, with a group size of 8 mice per group (n=8/group). In the preparation for tumor cell injection, the MDA-MB-231 cells were cultured until they reached a confluence of 70-90%. Subsequently, the growth medium was replaced with fresh medium 24 hours prior to harvesting through trypsinization. The cells inoculum comprising 0.3x10^6^ cells in 100 µl sterile PBS was injected subcutaneously at the right flank of the mice. The tumor volume was measured using Vernier calipers and calculated by using the formula 1/2 (Length x Width x Height). The tumor volume was taken bi-daily until the 21^st^ day. On the 21^st^ day post-implantation, the mice were sacrificed, and tumors from the respective xenografts were dissected for mechanistic studies. The weight was measured (n=4), followed by processing for hematoxylin and eosin (H&E) staining. For the H&E staining, tumor specimens were extracted and treated with 10% buffered formalin and embedded in paraffin for histological analysis to investigate the intra-tumoral proliferative compartments. A comparison was then made between the Myricetin and Myr-NE groups. Digital images of histology slides were observed using a bright-field microscope (IX 51, Olympus, Japan) with a magnification of 40x (objective) and 10x (eyepiece). Moreover, tumors from the different treatment groups and control were processed for the establishment of tumor-derived cells, and the rest of the tumor samples were snap-freezed in liquid nitrogen and then stored at -80°C for immunoblotting experiments.

### 2.7 Immunoblotting

The western blotting procedure of tumor tissue samples was carried out using the previously described method (19). Briefly, tumor tissues were lysed in RIPA lysis buffer, followed by centrifugation, protein isolation, and concentration estimation. 60-80 micrograms (μg) of proteins from each group were resolved on 8-15% SDS-PAGE gel (depending on the molecular weight of the proteins). Then, it was electro-blotted on the PVDF membrane (MDI) and blocked with 5% BSA for 1 hour, followed by incubation with primary antibodies (Antibody details are mentioned in the material and methods section). The membrane was washed in Tris-buffered saline supplemented with 0.1% Tween-20 (TBST), followed by incubation with the appropriate HRP-conjugated secondary antibody dilution (1:5000) for 2 h. Blots were washed with TBST and developed using ECL chemiluminescence detection reagent using the Luminescent image analyzer (ImageQuant LAS 500, Japan). Blot densitometry was performed using Image J software to measure the levels of phosphorylated proteins and other investigated proteins (as mentioned in respective figure legends). The obtained values were then normalized with the relevant levels of total proteins (un-phosphorylated) and the loading control (β-Actin). Finally, treatment-induced differential change in protein expression was estimated and presented as the relative change with respect to control.

### 2.8 Establishment of tumor-derived cells

Tumor-derived cells from xenografts were established using the previously described method (19). An aseptic technique was employed to dissect the tumors from each xenograft (n=4/group), which were subsequently macerated in sterile PBS and then passed through a 70µm cell strainer (Millipore). Later, the cell suspension that underwent filtration was subjected to three rounds of washing with PBS and centrifugation at 1500g for 10 minutes. The pellet was finally resuspended in a cell growth medium, transferred to 60 mm tissue culture dishes, and subsequently incubated in a CO_2_ incubator at 37°C. After 24 hours of cell incubation, further mechanistic studies were performed, and the treatment effect was compared with respect to control.

### 2.9 *In-vitro* wound-healing assay (scratch assay), and invasion assay

The cell migration experiment was performed according to the previously described protocol in tumor-derived cells (19). In brief, 0.006×10^6^ cells were seeded in 96 well plates and allowed to attain the confluency. A mechanical scratch was created by scraping using a sterile pipette tip (volume of 200 µl). As cells moved from the intact areas to the scratched region, images were captured at different intervals (0, 24, and 48h) using an inverted bright-field microscope (Olympus, Japan) with a 10 × 10 X magnification. Further quantitative analysis of migrated cells was performed on Image J software with appropriate scaling and migration potential presented as a time-dependent change in area coverage (%) by the respective tumor cell group. Likewise, the transwell invasion assay was carried out to examine the distinct chemotactic responses in tumor-derived cells from control, Myricetin, and Myr-NE xenografts using the previously described method (19). The Hanging Millicell inserts (Millipore) with 8-mm polyethylene terephthalate membrane filters were placed in 24 well plates. Cells were seeded in each upper chamber of the hanging insert with a density of 0.04×10^6^ cells (in serum-free media). In order to facilitate chemotactic movement, a complete growth medium supplemented with 10% FBS was added to a 24-well plate (facing the lower chamber of the insert) and incubated in a CO_2_ incubator for a duration of 24 hours. The cells in the upper chamber were wiped out using a cotton swab, while cells in the lower chamber were processed for fixation in paraformaldehyde (4%). Further, cells were permeabilized with methanol, and Giemsa staining was performed, followed by image acquisition under a bright field microscope (10x10X magnification). Finally, the images from the different fields were processed on image J software for quantification, and the number of invading cells was plotted as a relative tumor cell invasion in Myricetin and Myr-NE cells with respect to control.

### 2.10 Cell growth, clonogenic, and CFSE cell proliferation assay

Cell growth assay was performed in tumor-derived cells. Cells were seeded with the density of 0.03×10^6^ cells in a 24-well plate. Further cells were kept in a CO_2_ incubator, and real-time cell growth was monitored for up to 72 hours using Zencell Owl live imaging system (24-well plate camera-based system). Cell density with respective images was obtained from instrument software, and the graph plotted as the relative cell growth with respect to control. A clonogenic assay was performed in tumor-derived cells using the previously described method (20). Briefly, 100 cells from each group were plated into PD-60 and incubated for 7-8 days in a CO_2_ incubator to allow the development of macroscopic colonies. Further cells were washed with PBS, followed by staining with crystal violet. The colonies containing ≥50 cells were counted, and the percent clonogenicity was calculated using the formula (No. of colonies formed/No. of cell-seeded)×100, and the graph plotted as % clonogenicity of the respective group. According to the manufacturer’s protocol, Carboxyfluorescein succinimidyl ester (CFSE) cell proliferation kinetics were performed in tumor-derived cells from each xenograft (n=3). Cells were transferred to a 15ml falcon tube containing CFSE (5μM/ml in cell growth medium supplemented with 2% serum) and incubated at room temperature for 20 min (with continuous rolling). Further cells were seeded with the density 0.003×10^6^ in 96 black well plates, and time kinetics fluorescence measurement was performed at each interval of 24 h up to 72 h on a microplate reader (VANTAstar; BMG Labtech; Germany) using excitation and emission wavelength of 492/517nm. As cells divide with each successive cell division, the time-dependent reduction in CFSE fluorescence (from 0 to 72h) indicates an increase in cell proliferation and vice-versa. Finally, the obtained values at 24 to 72 h were normalized with initial respective 0hr readings, and the CFSE proliferation graph was plotted as percent fluorescent reduction, indicating the extent of cell proliferation in the individual tumor cell groups.

### 2.11 ROS, nuclear fragmentation, and Acridine orange/Ethidium bromide staining

An intracellular ROS was measured using CM-H2DCFDA staining in tumor-derived cells as per our previously described method (20). The nuclear fragmentation assay was performed in accordance with the methodology described in the reference (20, 21). The tumor-derived cells were seeded with the density of 0.075×10^6^ cells in PD35 containing coverslip and kept at 37°C in a CO_2_ incubator. Following a 24-hour incubation period, the cells were fixed in 4% paraformaldehyde solution for a period of 10 minutes at 37°C. A coverslip was placed on a microscopic glass slide using DAPI (4,6-di-amidino-2-phenylindole) mounting solution, and images were acquired on a fluorescence microscope (Olympus IX 51 fluorescence microscope, Japan) under a UV excitation channel (40×10 X magnification). For Acridine orange/Ethidium bromide (AO/EB) staining, tumor-derived cells were seeded with a density of 0.075×10^6^ cells in PD35. At 48 h. cell growth medium was transferred in a 15ml falcon tube to ensure the collection of dead cells in floaters. Further cells were trypsinized and transferred to the respective falcon tube, followed by centrifugation at 100g for 10 minutes. The pellet was resuspended in 500μl PBS, transferred to 96 well plates, and re-centrifuged at 100g to allow the cells to settle down. Finally, 20μl of AO/EB stain solution (100μg/ml; in the ratio of 1:1) was added to each well, and fluorescence imaging was performed on a microscope (Olympus IX 51 fluorescence microscope, Japan) under blue and green excitation channels (10×10 X magnification) for AO and EB, respectively. AO/EB (live/dead) positive cells were enumerated using microscope software, and the graph was plotted as the number of live versus dead cells in respective tumor cell groups.

### 2.12 Statistical analysis

Statistical comparisons between the experimental groups were performed using one-way ANOVA (analysis of variance) with the Tukey multiple comparison test on the GraphPad Prism software (version 10). Data are presented as the mean ± SD, and statistical significance was determined for differences between means at a significance level of *P* ≤ 0.05.

## 3 Results

### 3.1 Myr-NE inhibits tumor growth in TNBC xenografts

**Figure 1:**
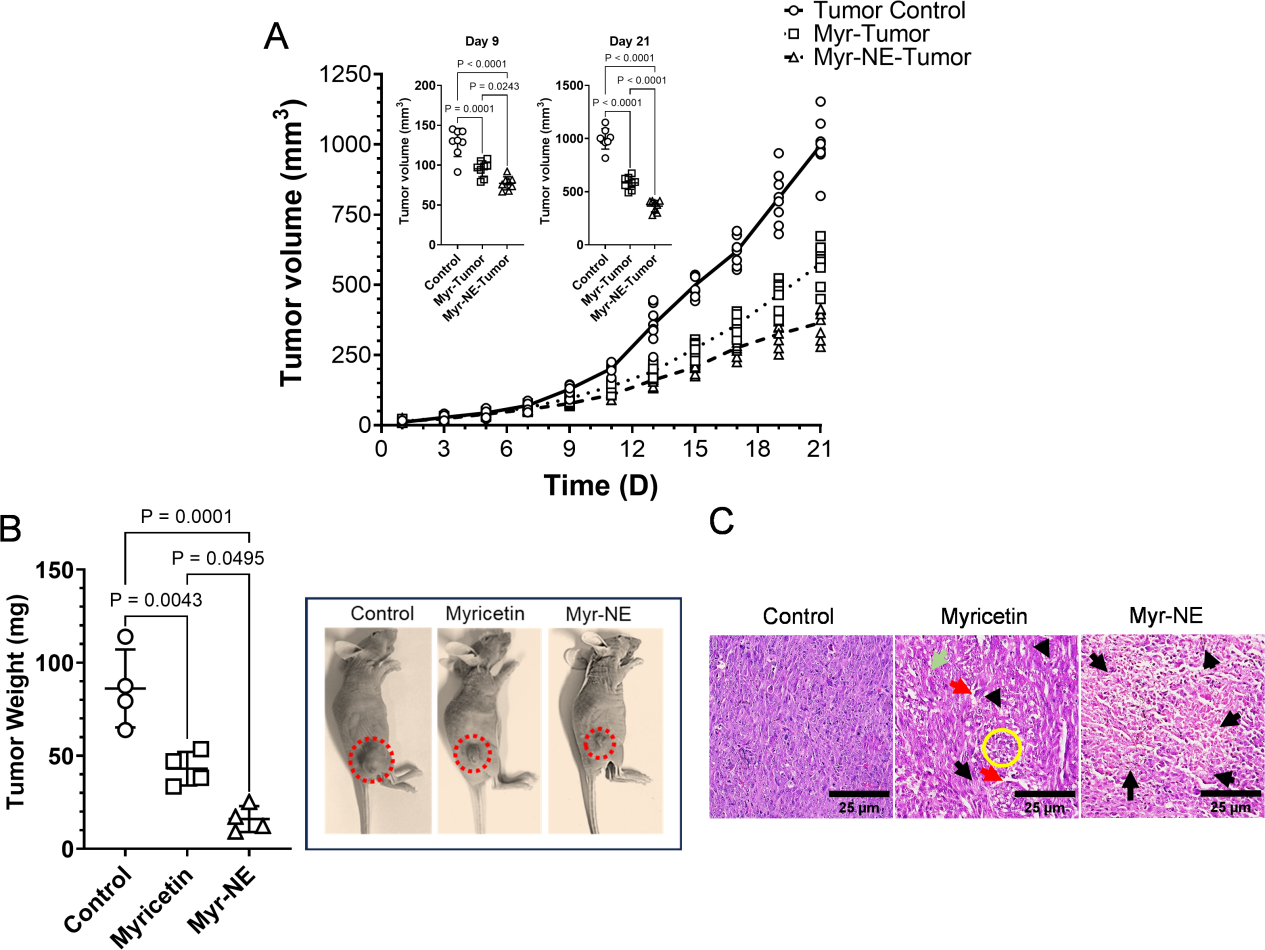
Effect of myricetin and Myr-NE treatment on tumor proliferation. **(A)** The tumor proliferation graph indicates the differential change in tumor volume with respect to time (days-’D’) in the indicated experimental groups (n=8/group) of TNBC xenografts. An inset scatter plot shows the significant difference in tumor volume following Myricetin and Myr-NE treatment at the 9^th^ and 21^st^ day post-tumor implantation. **(B)** Graph indicating the tumor weight (n=4; day 21). The representative image of indicated TNBC xenografts points to the visual change in tumor burden on the 21^st^ day post-tumor implantation. Additional sets of tumor xenograft images are given in the **Supplementary Figure**. **(C)** Photomicrographs show the histological analysis of tumor tissues following H&E staining at the 21^st^ day post-tumor implantation. Arrow marking indicated as extravasation (red arrow), endothelial damage and blood cells (green arrow), early-stage tumor shrinkage, small, fragmented nucleus (yellow circle), and late-stage necrosis (black arrow). Data represents the mean ± SD, and statistical significance was calculated between the experimental groups, mentioned as *P* ≤ 0.05.

We established TNBC xenograft models to examine the anti-tumor efficacy of Myr-NE and compared it with Myricetin treatment. Tumors were formed with MDA-MB-231 cells (0.3×10^6^) implantation in three different groups of Control, Myricetin, and Myr-NE (n=8/group). The intraperitoneal administration of Myricetin (50mg/kg body weight) and Myr-NE (25mg/kg body weight) was started on the 5^th^ day of post-tumor implantation and continued for seven days. In the Myr-NE treated xenograft, the significant difference in tumor volume was noted on the 9^th^ day (4^th^-day post-treatment) as compared to Myricetin and remained persistent up to the 21 days post-tumor implantation (**Figure 1A** inset scatter plot). By the 21^st^ day, the average tumor volume of control xenografts reached to 996.75 ± 84.83 mm^3,^ which was significantly reduced to 583.31 ± 53.76 mm^3^ (~42%) and 364.78 ± 47.65 (64%) following Myricetin and Myr-NE treatment respectively. (**Figure 1A**). It was intriguing to observe that administering a half dose of Myr-NE (25 mg/kg) showed promising results in significantly reducing tumor proliferation or growth compared to Myricetin (50 mg/kg) treatment. The extent of reduction in tumor growth was further correlated with the tumor weight of both the treatment groups and compared with the control. The average tumor weight of the control, Myricetin, and Myr-NE treatment were estimated as 86.14 ± 18.15 mg, 43.04. ± 6.96 mg and 15.98 ± 5.4 mg, respectively (**Figure 1B**). A significant reduction in the tumor burden was observed in Myr-NE treated groups compared to Myricetin-treated xenografts (**Figure 1B**). The representative images depict the changes in the gross tumor burden of xenografts (**Figure 1B**). Further examination of the intratumor microenvironment using Hematoxylin and Eosin staining revealed that the control tumor has polygonal and spindle-shaped cells and dense neoplastic tissue features, indicating the advanced stage of TNBC (22) (**Figure 1C**). Following Myricetin treatment, the tumor showed early-stage tumor shrinkage and late-stage necrotic area (ghost cells with light or no nuclear stain). At the same time, tumors from Myr-NE xenografts observed with extensive necrosis evidenced by a high number of ghost cells with minimal hematoxylin staining suggested the substantial tumor cell death surpassing the effect of Myricetin against TNBC (**Figure 1C**).

These results demonstrate that Myr-NE treatment significantly reduces the rate of TNBC proliferation and enhances tumor cell death compared to Myricetin alone.

### 3.2 Myr-NE attenuates PAM signaling activation and downregulates tumorigenesis-associated proteins in TNBC xenografts

**Figure 2:**
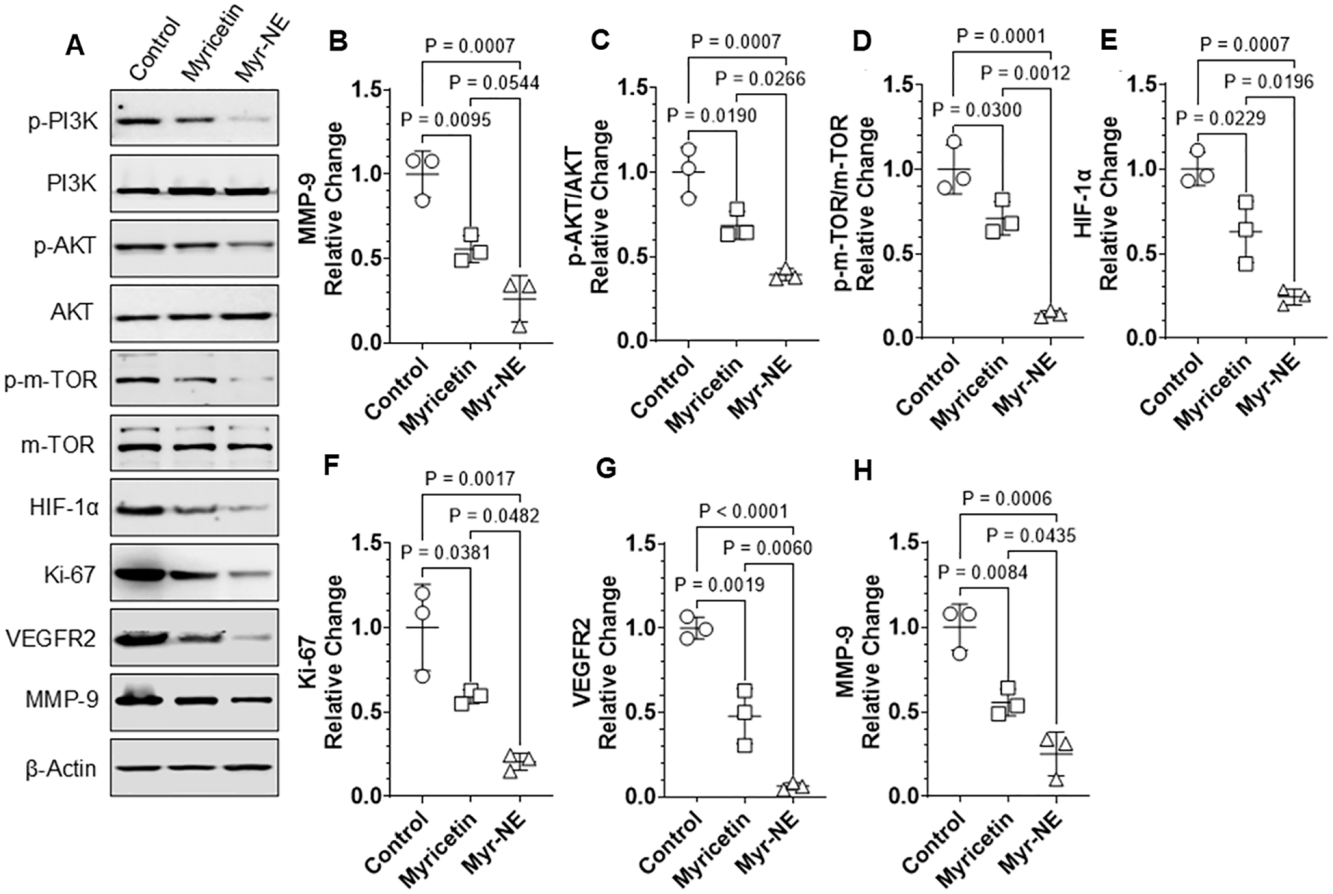
Myr-NE-induced inhibition of PAM signaling and tumorigenesis-associated proteins. **(A)** Tumors were dissected on the 21^st^ day post-tumor implantation, and immunoblotting was performed in tumor lysates (n=3) to examine indicated proteins (additional blot images (n=3) are provided in the **Supplementary Figure 1**. **(B–H)** Scatter plots indicate the relative change in the indicated protein expression with respect to the control. Data represents the mean ± SD and statistical significance between the experimental groups mentioned as *P* ≤ 0.05.

Multiple studies demonstrate that within the realm of cancer, the abnormal activation of PI3K/AKT/mTOR (PAM) signaling promotes tumor survival and proliferation (23). Like other subtypes of breast cancer, the PAM pathway is frequently altered in TNBC, supporting the notion that targeting these signaling cascades may provide better clinical outcomes (24). Therefore, we sought to investigate the distinct impact of Myricetin and Myr-NE on PAM signaling pathways, which, in turn, influenced the differential rate of tumor proliferation in TNBC xenografts. On the 21^st^ day of post-implantation, tumors were dissected and subjected to immunoblotting. A substantial downregulation of the PI3K/AKT/mTOR phospho-proteins was observed in the tumor cell lysate of Myricetin-treated xenografts, compared to the control (**Figures 2A–D**). This result was consistent with the earlier reported observations that Myricetin possesses the ability to inhibit the activation of the PAM pathway, hence disrupting the downstream signaling pathways that promote tumor formation (10). However, tumor samples from Myr-NE-treated xenografts exhibited notable downregulation of PAM signaling, as evidenced by the substantial decrease in the phospo-PAM proteins, compared to Myricetin-treated xenografts. (**Figures 2A–D**). These results suggested the nanoemulsion-mediated enhanced effect of Myricetin on anti-tumor signaling in TNBC xenografts. To validate these observations, the subsequent analysis focused on the examination of other key factors involved in tumorigenesis, including hypoxia-inducible factor 1 alpha (HIF-1α), tumor proliferation marker protein Ki67, vascular endothelial growth factor receptor 2 (VEGFR2) and matrix metalloproteinase (MMP-9). The expression of all these proteins was considerably reduced in the Myricetin-treated xenografts as compared with control. Whereas, as compared to Myricetin treatment, a further significant attenuation in all the tumorigenic proteins was observed following Myr-NE treatment, confirming its potential anti-tumor activity in TNBC xenografts (**Figures 2A, E–H**).

Together, these results suggest that Myr-NE treatment results in efficient inhibition of PAM proteins and VEGFR2 accompanied by significant downregulation of HIF-1α, Ki67, and MMP9 proteins, effectively reducing the tumor proliferation in TNBC xenografts.

### 3.3 Myr-NE tumor-derived cells exhibit reduced TNBC proliferation and clonogenicity

**Figure 3:**
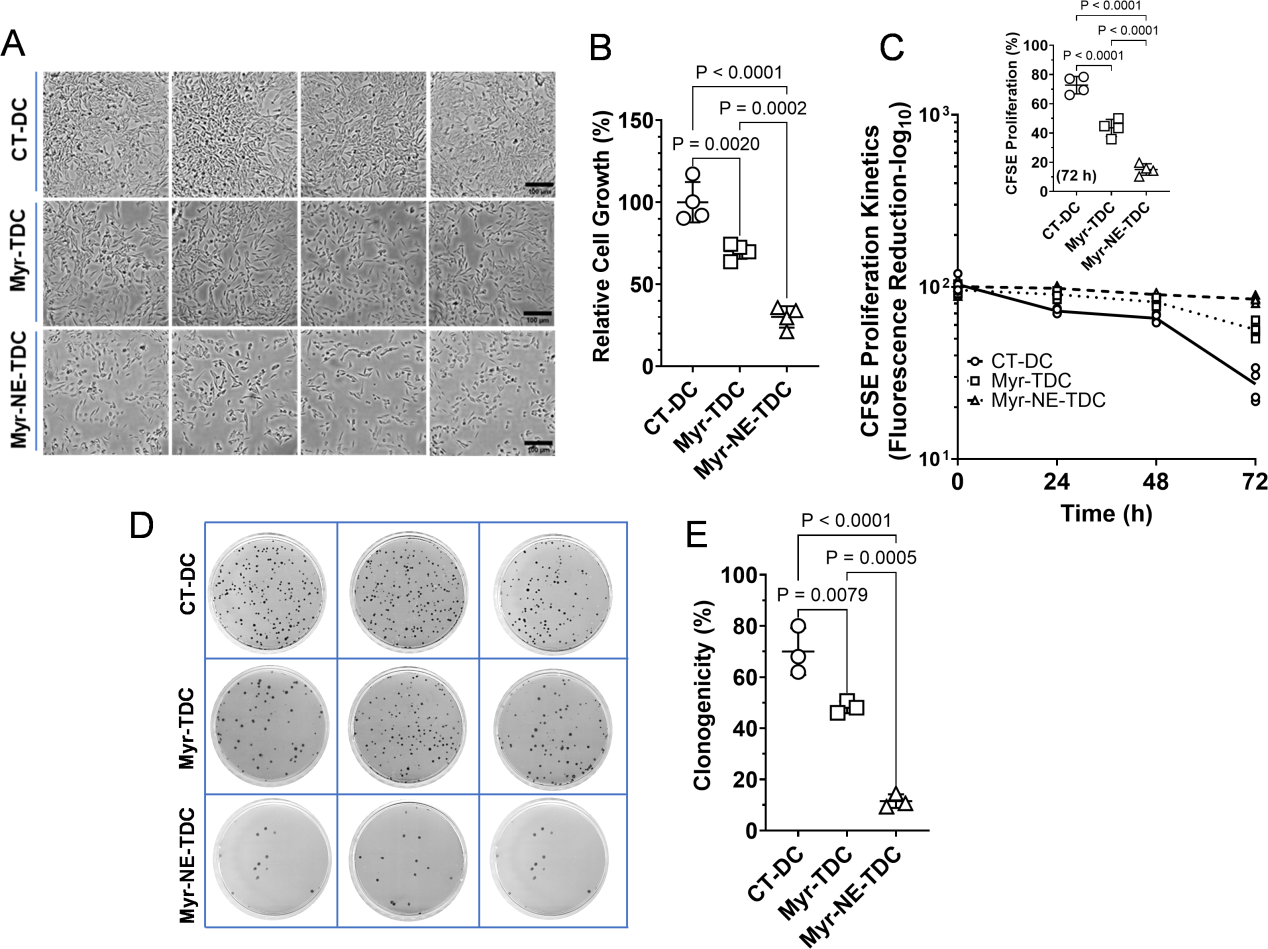
Tumor-derived cells of Myr-NE xenograft exhibited reduced cell proliferation and clonogenicity. **(A)** The representative micrographs of cell growth in tumor-derived cells of TNBC xenografts presented at 72 h post cell seeding in the CT-DC (tumor cells derived from control xenografts), Myr-TDC (tumor cells derived from Myricetin treated xenografts), and Myr-NE-TDC (tumor cells derived from Myr-NE treated xenografts) groups. **(B)** The scatter plot shows the quantitative estimation of cell growth at 72 hours (h) in the indicated groups (n=4). **(C)** The line graph illustrates the kinetics of CFSE proliferation at 24, 48, and 72 hours (h), and the decrease in time-dependent CFSE fluorescence corresponds to an increase in cell proliferation of tumor-derived cells (n=4). The inset scatter plot indicates the change in tumor cell proliferation (derived information) at 72 h post-tumor cell seeding. **(D)** The representative images of Petri-dishes display the differential number of colonies in the indicated groups (n=3). **(E)** The scatter plot shows the percent change in clonogenicity of tumor-derived cells. Data represents the mean ± SD and statistical significance between the experimental groups mentioned as *P* ≤ 0.05.

To investigate if the differential effect of Myricetin and Myr-NE is also retained with time in xenograft tumor-derived cells, we examined the tumor cell growth, cell proliferation, and clonogenicity and compared them with control xenograft tumor-derived cells. A significant reduction in cell number was observed in both the treatment tumor-derived cells compared to the control group (n=4). However, a notable decrease in cell number was noted in the Myr-NE when compared to the Myricetin tumor-derived cells (**Figures 3A, B**). The cell growth assay was further correlated with the CFSE cell proliferation kinetics. Tumor cells from control xenografts showed a time-dependent significant decrease in CFSE fluorescence at 24, 48, and 72h. indicated the progressive increase in cell number compared to both the treatment tumor-derived cells (**Figure 3C**). At the same time, the inhibition effect of Myr-NE on the growth of tumor cells remained consistent throughout all the examined time intervals and exhibited a noteworthy reduction compared to Myricetin tumor-derived cells (**Figure 3C**). To validate the results of tumor cell growth and proliferation, the clonogenic potential was examined in all the tumor-derived cells. The clonogenicity of control was observed as 70 ± 7.48%, which considerably reduced to 48.22 ± 1.91% and remained at only 11.5 ± 2.19% in Myricetin and Myr-NE tumor-derived cells, respectively (**Figures 3D, E**). These results were significantly correlated with tumor cell growth and proliferation and suggestive of the retained anti-tumor efficacy of Myr-NE over and above Myricetin treatment.

Together, these results suggest that the anti-TNBC activity of Myr-NE is sustained in tumor-derived cells, which surpasses the effect of Myricetin alone.

### 3.4 Tumor cells derived from Myr-NE xenografts showed reduced cell migration and invasion

**Figure 4:**
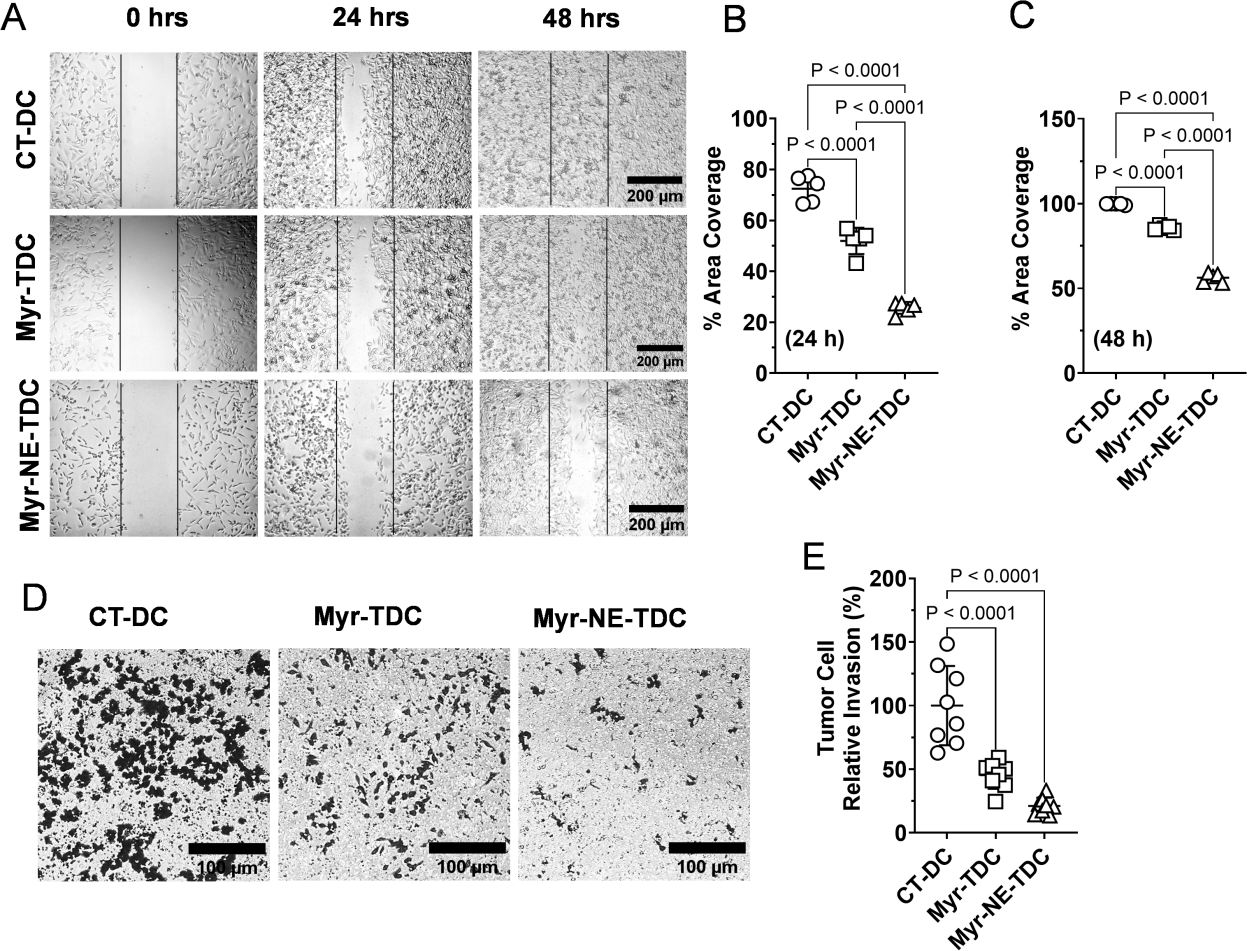
Inhibition effect of Myr-NE on the migration and invasion potential. **(A)** Photomicrographs depicting the migration potential of tumor-derived cells at the indicated time points. **(B, C).** The scatter plot shows the quantitative estimation of the number of migrated cells at 24 **(B)** and 48 h **(C)**, obtained after normalizing the respective initial values (0-h) and presented as percent area coverage with respect to time (n=3). **(D)** The representative image shows the invasion potential of tumor-derived cells from control (CT-DC), myricetin-treated (Myr-TDC), and Myr-NE-treated (Myr-NE-TDC) xenografts. **(E)** The scatter plot indicates the relative invasion potential of Myricetin and Myr-NE tumor cells with respect to control. Data represents the mean ± SD and statistical significance between the experimental groups mentioned as *P* ≤ 0.05.

To substantiate our hypothesis of better Myr-NE effectiveness for TNBC treatment, we sought to observe the tumor cell migration and invasion potential compared with Myricetin tumor-derived cells. A mechanical scratch wound was created in the tumor-derived cells for cell migration. The migration potential in terms of area coverage of tumor control was estimated as 72.45 ± 4.67% (24 h) and attained confluency (~100 ± 0.37%) at 48h, indicating the aggressive characteristic of TNBC tumor cells (**Figures 4A–C**). On the contrary, tumor cells derived from Myricetin-treated xenografts showed a considerable reduction in the migration potential estimated as 52 ± 4.6% (24h) and 85.62 ± 1.16% (48 hrs). Consistent with earlier results, the Myr-NE tumor-derived cells exhibited a significant decrease in TNBC migration potential, which remained at 26 ± 2.09% and 56 ± 2.44% at 24 and 48 h, respectively. This indicates a strong inhibitory effect of Myr-NE over Myricetin at both tested time points (**Figures 4B, C**). These results were further validated by examination of tumor cell invasiveness using the Boyden chamber assay. Tumor-derived cells from control xenografts showed a considerable increase in the chemotactic movement compared to both the treatment tumor-derived cells (**Figure 4D**). However, Myr-NE xenografts-derived tumor cells showed a significant reduction in invasion potential estimated as 21.13 ± 6.06% as compared to 45.29 ± 10.07% in tumor cells derived from Myricetin xenograft (**Figure 4E**).

The results suggest that Myr-NE treatment significantly inhibits the migration and invasion potential of TNBC tumor cells.

### 3.5 Myr-NE treatment resulted in enhanced tumor cell death by inducing oxidative stress and DNA damage

**Figure 5:**
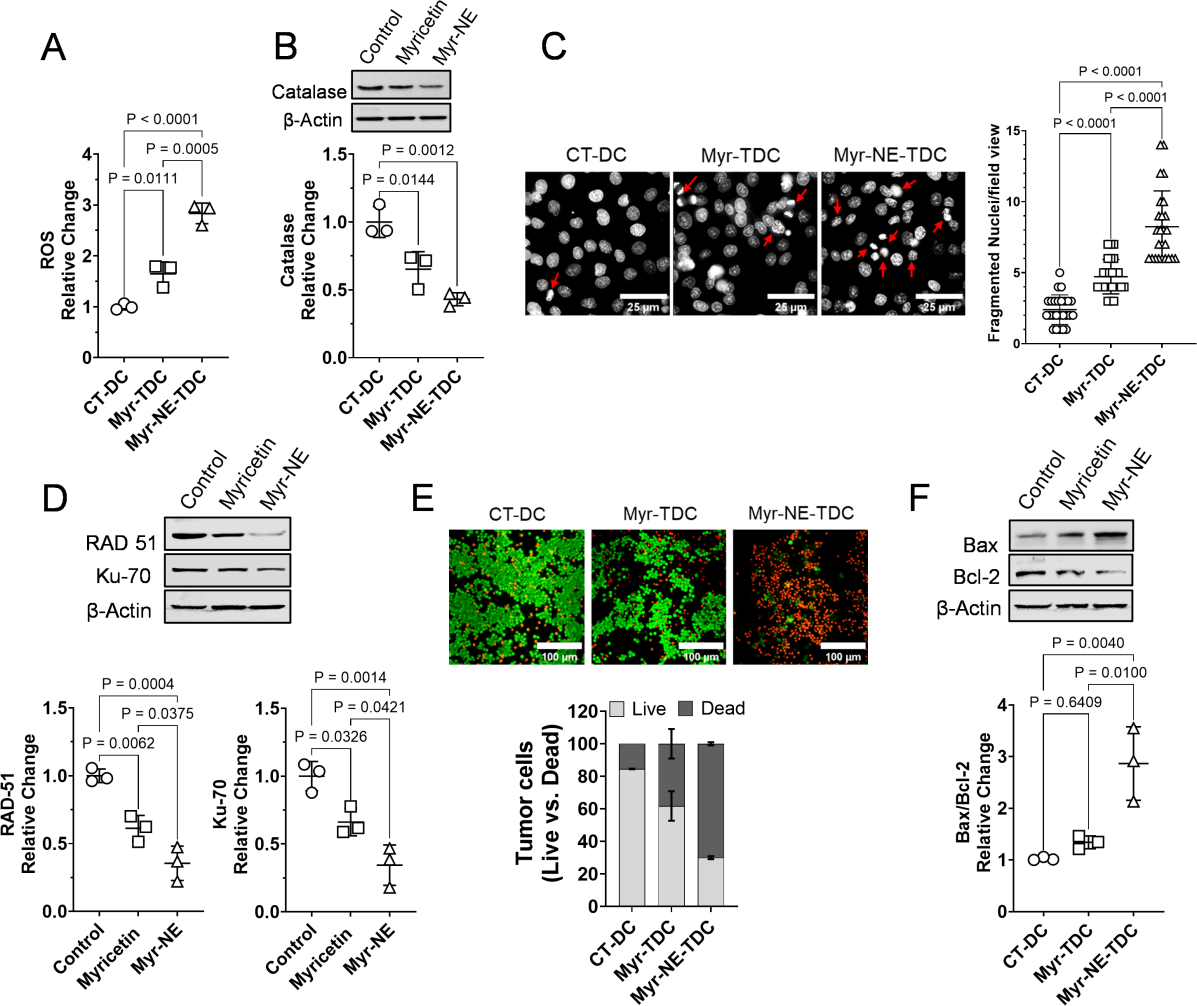
Myr-NE treatment-induced oxidative stress and DNA damage leads to enhanced tumor cell death. **(A)** The scatter plot indicates the Myricetin and Myr-NE induced relative change in intracellular ROS level with respect to control in tumor-derived cells (n=3). **(B)** The immunoblotting was carried out in whole tumor lysate of TNBC xenografts for the examination of antioxidant protein catalase, and the graph of densitometric evaluation shows the relative change in protein expression with respect to control. Additional blot images (n=3) are provided in the **Supplementary Figure 2**. **(C)** Nuclear fragmentation assay was performed in tumor-derived cells. The representative photomicrographs show the nuclear morphology (greyscale) with arrows indicating the fragmented nuclei in tumor-derived cells. The adjacent graph shows the number of fragmented nuclei in the different xenograft (n=4) derived cells; data was obtained from the different microscopic field views (25) of tumor cells. **(D)** Immunoblot of the tumor tissue samples and densitometric evaluation of the indicated DNA repair proteins presented as the relative change with respect to control (n=3; additional blot images are provided in the **Supplementary Figure**). **(E)** The representative photomicrographs indicate the ingress of AO/EB staining, whereas the below bar plot indicates the corresponding ratio of live versus (vs.) dead cell population as AO vs. EB positive cells, respectively. **(F)** Immunoblot indicating the expression of pro-apoptotic (Bax) and antiapoptotic (Bcl2) proteins in tumor tissue samples of TNBC xenografts. The scatter plot indicates the relative change in the Bax/Bcl2 ratio with respect to control (n=3; additional blot images are provided in the **Supplementary Figure**). Data represents the mean ± SD and statistical significance between the experimental groups mentioned as *P* ≤ 0.05.

To this end, we investigated the cell proliferation, clonogenicity, migration, and invasion of tumor cells derived from TNBC xenograft. We sought to gain intracellular insight into the tumor cells by analyzing oxidative stress, DNA damage, and cell death. It is evident that tumor cells tend to increase ROS production, which promotes tumor cell proliferation; however, excessive ROS generation leads to cell death (25). Myricetin is known to have prooxidant activity in tumor cells (26). In line with these observations, a notable increase in ROS production was estimated as 1.64 ± 0.189 fold in Myricetin xenografts derived tumor cells, which further significantly increases to 2.83 ± 0.165 fold in Myr-NE tumor cells, compared to control (**Figure 5A**). These results were also correlated with the antioxidant enzyme level, i.e., catalase in tumor tissue samples of xenografts. A considerable inhibition in catalase by 34% and further significant attenuation of 56% were observed in tumor tissue of Myricetin and Myr-NE-treated xenografts, respectively, compared to control (**Figure 5B**). We additionally intend to examine the consequence of increased oxidative stress in tumor cells of both treatment groups. Excessive ROS generation is well-reported to cause DNA damage in tumor cells (27). Consistent with these findings a significant alteration in the redox status of Myr-NE tumor-derived cells was found in association with a notable increase in DNA fragmentation when compared to Myricetin tumor-derived cells (**Figure 5C**). These observations were validated by the status of DNA repair proteins of both homologous recombination (RAD51) and non-homologous end joining (Ku70) repair pathway in tumor tissue samples obtained from TNBC xenografts. The Myr-NE tumor xenografts showed a notable decrease in RAD51 and Ku70 proteins compared to Myricetin. These results indicated that in the tumor samples of Myr-NE treated xenografts, DNA repair is significantly impaired, leading to increased DNA damage compared to the Myricetin xenografts (**Figure 5D**). Next, we examined tumor cell death using Acridine orange (AO) and ethidium bromide (EB) staining in tumor-derived cells. An increased proportion of intracellular EB ingress in Myr-NE tumor-derived cells is suggestive of increased cell death as compared to Myricetin tumor cells (**Figure 5E**). Moreover, Myr-NE tumor samples showed a significant increase in Bax/Bcl2 ratio by 2.86 ± 0.58 fold, which confined to 1.34 ± 0.101 fold in Myricetin tumor, compared with control further confirming the enhanced activation of pro-apoptotic proteins in Myr-NE tumor cells (**Figure 5F**).

These findings suggest that Myr-NE treatment induced oxidative stress, DNA damage, and downregulation of the DNA repair mechanism, eventually contributing to enhanced tumor cell death in Myr-NE xenografts.

## 4 Discussion

The tumor microenvironment (TME) of TNBC is highly heterogeneous and significantly influences tumor initiation, proliferation, immune evasion, angiogenesis, invasion/cell migration, and poor prognosis (28, 29). The rapid progression and poor differentiation are the characteristic nature of TNBC tumors, resulting in a greater propensity for metastasis and recurrence, hence resistance to conventional chemotherapies (30). In recent years, the PI3K/AKT/mTOR pathway has been identified as a promising target for addressing drug resistance (3, 31). The dysregulation of this pathway is strongly linked to the progression of tumors and the development of resistance to standard therapies in breast cancer (32). Myricetin, a naturally occurring flavonoid, has demonstrated significant anti-cancer properties via targeting PI3K/AKT/m-TOR signaling cascade (10). However, the application of Myricetin is limited because of poor aqueous solubility, low bioavailability, permeability, and stability, limiting its effectiveness as a chemotherapeutic drug (33). We aimed to develop a myricetin-loaded nanoemulsion (Myr-NE) to overcome this challenge and carried out a differential efficacy evaluation study using TNBC models. Nanoemulsions are advantageous in overcoming solubility limitations. Additionally, nanoemulsion facilitates increased drug uptake, regulates drug release, and is a highly efficient strategy for improving therapeutic efficacy in TNBC treatment (13, 34–41). In our recent study, we compared the efficacy of Myr-NE with Myricetin alone in TNBC cells. The study provided considerable results, demonstrating that nanoemulsion enhances solubility and intracellular internalization, thereby improving the anti-TNBC activity of Myricetin in MDA-MB-231 cells (13). The present follow-up pre-clinical investigation aimed to examine the potential anti-tumor activity of Myr-NE in TNBC xenografts. While various research studies have documented the anti-cancer properties of Myricetin in various malignancies, its evidence in TNBC is limited and primarily confined to *in-vitro* cell lines (42). This *in-vivo* investigation also provides vital insights and strengthens the pre-existing notion about the potential role of Myricetin for TNBC treatment.

The administration of Myricetin led to a notable decrease in the tumor volume and tumor burden of TNBC xenografts, compared to control, thereby confirming its significance in TNBC treatment. However, compared to Myricetin alone, a significant decrease in tumor volume and tumor burden following Myr-NE treatment further confirmed its anti-TNBC activity over and above Myricetin (**Figure 1A**). Notably, a reduction shift in tumor volume from 1.73-fold (Myricetin group) to 2.7-fold (in Myr-NE groups) and a reduction by 2-fold to 5.38-fold in tumor burden was observed, respectively, at a dose two times lower than Myricetin (**Figures 1A, B**). Therefore, it is reasonable to speculate the enhanced anti-tumor efficacy of Myr-NE is most likely attributed to increased drug solubility and bioavailability gained by nanoemulsion (43). The observed difference in tumor proliferation led us to investigate the effect of both treatments on anti-tumor signaling in TNBC xenografts. Previous studies reported that the PAM signaling pathway is commonly altered across various subtypes of breast cancer, and its activation contributes to an enhanced rate of tumor proliferation, migration, and invasion in TNBC cells (24, 44, 45). In line with these observations, control xenografts showed increased phosphorylation of PAM proteins correlated with the accelerated tumor growth rate in TNBC xenografts. This effect was significantly reversed by Myricetin treatment, whereas the Myr-NE showed an efficient attenuation in the phosphorylation of PAM proteins, eventually contributing to reduced tumor growth as compared to Myricetin-treated xenografts (**Figures 1**, **2A–D**). PAM signaling-mediated overexpression of ATP-binding cassette (ABC) transporters facilitates the ATP-dependent removal of different chemotherapeutic drugs from cellular membranes, significantly contributing to TNBC chemoresistance (46, 47). Consequently, our study additionally indicates the potential role of Myricetin in overcoming this resistance in TNBC by inhibiting PAM signaling (**Figures 2A–D**).

Among the different receptor tyrosine kinases (RTKs), VEGFR2 is reported to act as a key activator of PI3K/AKT signaling, which is important for tumor survival. The binding of a ligand to VEGFR-2 activates PI3K and then activates PI3K phosphorylates PIP2, leading to the reactivation of PIP3, which subsequently triggers the PI3K/Akt signaling pathway (23). Earlier studies reported that VEGFR2-mediated activation of the PAM signaling pathway is essential for tumor survival, and Myricetin is known to inhibit the growth of breast tumor cells by regulating VEGF (23, 48, 49). Consistent with these findings, we observed that Myricetin exerts its inhibitory effects on the PAM pathway by downregulating VEGFR2, and the inhibition efficacy is further enhanced by Myr-NE treatment (**Figures 2G**). These findings add to the potential advantage of Myr-NE in the efficient inhibition of both VEGFR2 and PAM signaling, which is believed to be an effective targeted strategy for TNBC treatment (23, 50, 51). VEGFR2-induced activation of the PI3K/Akt pathway stimulates the release of MMP9 and promotes tumor cell invasion and migration in TNBC cells (52, 53). A considerable reduction in the MMP-9 protein expression was observed following Myr-NE treatment, compared to Myricetin (**Figure 2H**). The treatment-induced change in the MMP9 was further correlated with the significant reduction in the migration and invasion potential of Myr-NE xenograft-derived tumor cells as compared to Myricetin (**Figure 4**). This difference is most likely ascribed to how both treatments affected PAM signaling in TNBC xenografts, thereby confirming the advantage of Myr-NE over Myricetin (**Figures 2A–D**). Considering the fact that PAM signaling plays a crucial role in regulating HIF-1α, which is significantly upregulated in 80% of TNBC cases and is associated with elevated levels of glycolysis, stemness, angiogenesis, invasion, metastasis, and immune evasion, thus promoting the survival of TNBC (54). Our findings showed an efficient inhibition of PAM signaling following Myr-NE treatment, further resulting in the substantial reduction of HIF-1α, confirming its potential efficacy over and above Myricetin treatment against TNBC (**Figure 2E**). The overexpression of HIF-1α contributes to increased tumor proliferation by elevating the level of Ki67 in breast cancer (52, 53). The tumor obtained from Myr-NE treated xenografts also showed substantial attenuation of Ki67 compared to Myricetin treatment group, confirming the effective downregulation of HIF-1α in TNBC xenografts (**Figures 2E, F**) (32). In addition to the accelerated rate of tumor proliferation, the upregulation of Ki67 is also associated with increased invasiveness and proposed as a prognostic marker for unfavorable outcomes in TNBC patients (55). We observed significantly reduced tumor proliferation in both Myr-NE xenografts and its tumor-derived cells compared to Myricetin-treated xenografts, which correlated with the efficient downregulation of Ki67 following Myr-NE treatment (**Figures 2A, F**).

Mounting evidence suggests that Myricetin induces cell death by increased oxidative stress and DNA damage in tumor cells (56, 57). Our further investigation of the tumor oxidative stress revealed that Myr-NE treated tumor cells have significantly increased levels of intracellular peroxide (H_2_O_2_), compared to tumor cells derived from Myricetin xenograft (**Figure 5A**). These results were validated by the notable decrease in the antioxidant protein catalase of Myr-NE tumor, compared to Myricetin-treated tumor xenografts (**Figure 5B**). Increased oxidative stress in Myr-NE tumors was correlated with the effective inhibition of HR and NHEJ DNA repair proteins (27) (**Figure 5D**). Previous studies have indicated the role of PI3K/AKT signaling in DNA repair, which impairs the effectiveness of anti-cancer drugs and promotes therapeutic resistance (58, 59). Significant downregulation of the Rad-51 and Ku70 proteins following Myr-NE treatment is most likely attributed to the effective inhibition of PI3K/AKT signaling as compared to Myricetin in TNBC xenografts (**Figures 2A–D**). The downregulation of DNA repair proteins suggests a higher occurrence of double-strand breaks and DNA damage in Myr-NE tumors compared to Myricetin. This effect was evidenced by the substantial increase in nuclear fragmentation, which eventually led to enhanced cell death in Myr-NE tumor cells as compared to tumor-derived cells from Myricetin xenografts (**Figure 5C**). Consistent with our earlier reported observations, enhanced tumor cell death was validated by the significantly increased Bax/Bcl2 ratio in tumor tissue of Myr-NE xenografts, compared to Myricetin treated xenografts (**Figure 5F**) (13).

The present study was conducted over a 21-day period (for tumor proliferation) as a proof-of-concept to evaluate the enhanced therapeutic efficacy of Myr-NE compared to Myricetin alone. Consequently, a key limitation is that long-term efficacy, tumor recurrence, and sustained safety could not be assessed within this timeframe which warrants follow-up investigation. Additionally, pharmacokinetic data on absorption, distribution, metabolism, and excretion (ADME) of Myr-NE are essential to determine the optimal dosing range, its bioavailability, and therapeutic potential. Our subsequent study will investigate the comprehensive ADME analysis of Myr-NE, to strengthen the translational relevance of these findings and further validate nanoemulsion as a promising drug delivery strategy for TNBC treatment.

## 5 Conclusion

In conclusion, our pre-clinical investigation demonstrates that the utilization of nanoemulsion augmented the anti-TNBC activity of Myricetin by efficient inhibition of VEGFR2 and PAM proteins, leading to downregulation of HIF-1α, KI67, and MMP-9 proteins in TNBC xenografts. Moreover, increased oxidative stress and downregulation of DNA repair proteins following Myr-NE treatment contribute to increased DNA damage and tumor cell death in TNBC xenografts. Therefore, these results strengthen the notion that nanoemulsion potentiates the anti-tumor activity of Myricetin attributed to improved aqueous solubility and bioavailability, thereby providing a promising drug-delivery strategy for TNBC treatment and better clinical outcomes.

## Data Availability

The original contributions presented in the study are included in the article/**Supplementary Material**. Further inquiries can be directed to the corresponding authors.
