# Supplementary material for: Nanoemulsion of myricetin enhances its anti-tumor activity in nude mice of triple-negative breast cancer xenografts
Source: Front Oncol. 2025 Jun 9;15:1563076. doi: 10.3389/fonc.2025.1563076 (PMC12183182; doi:10.3389/fonc.2025.1563076)
Supplement: Supplementary file 1 [file DataSheet1.pdf]

**Supplementary Figure 1**

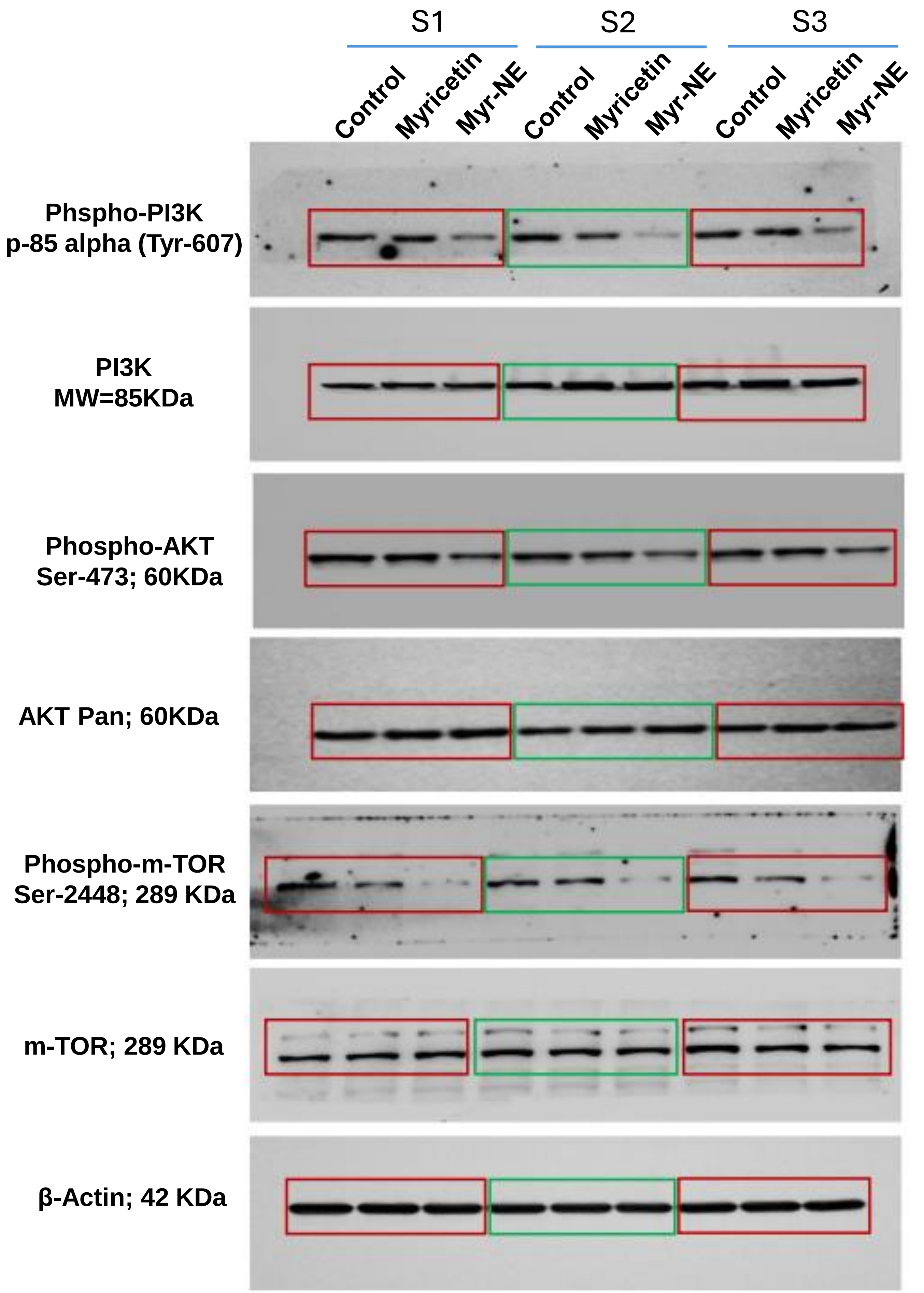

# Supplementary Figure 1 Continue

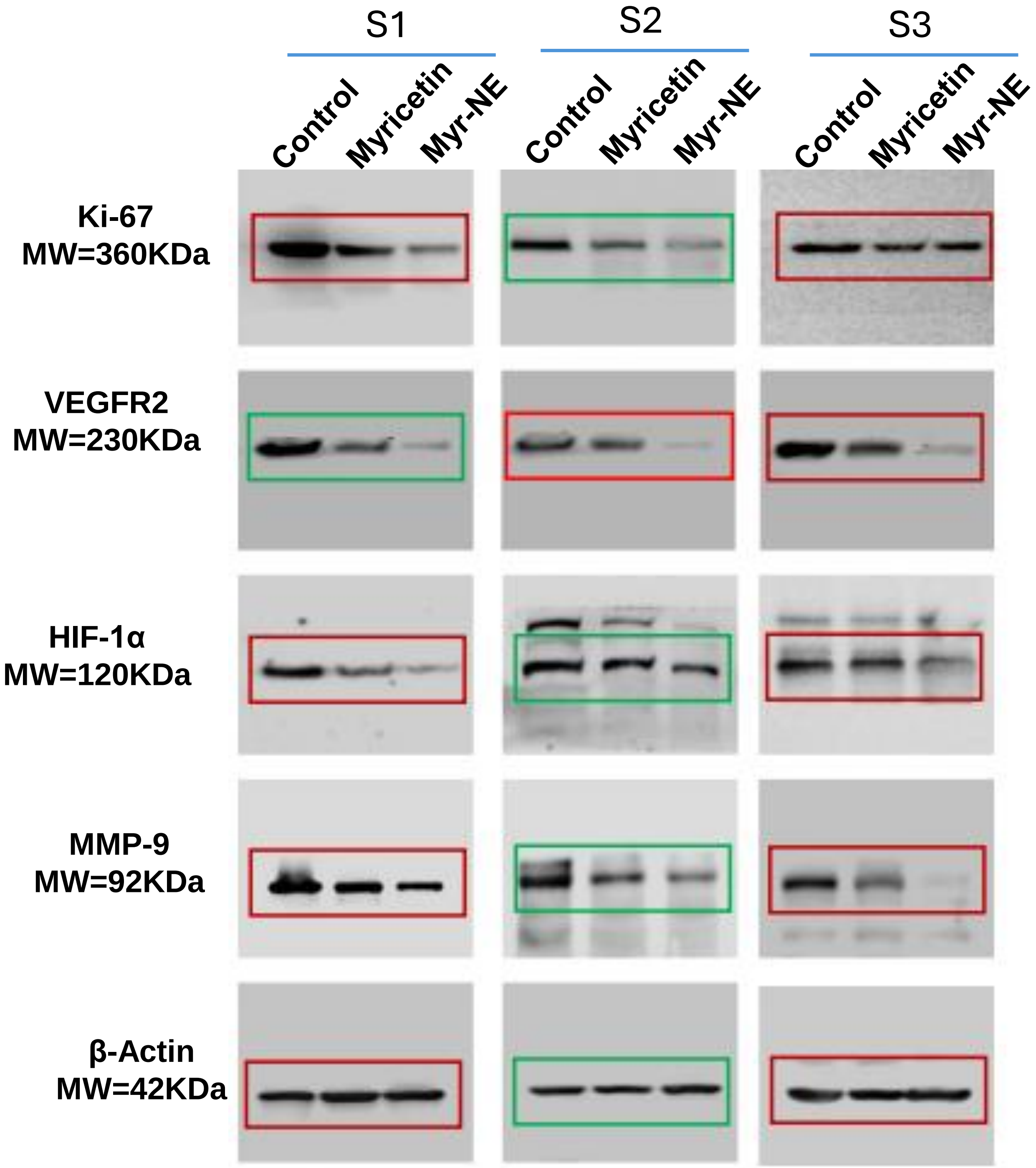


**Supplementary Figure 1.** Three blot panels of indicated proteins presented with three independent sets of tumor samples obtained from Control, Myricetin, and Myr-NE tumor xenografts (n=3). Green outline blot images of all the indicated proteins are presented in the main figure **(Fig.2A)** of the manuscript.

## Supplementary Figure 2

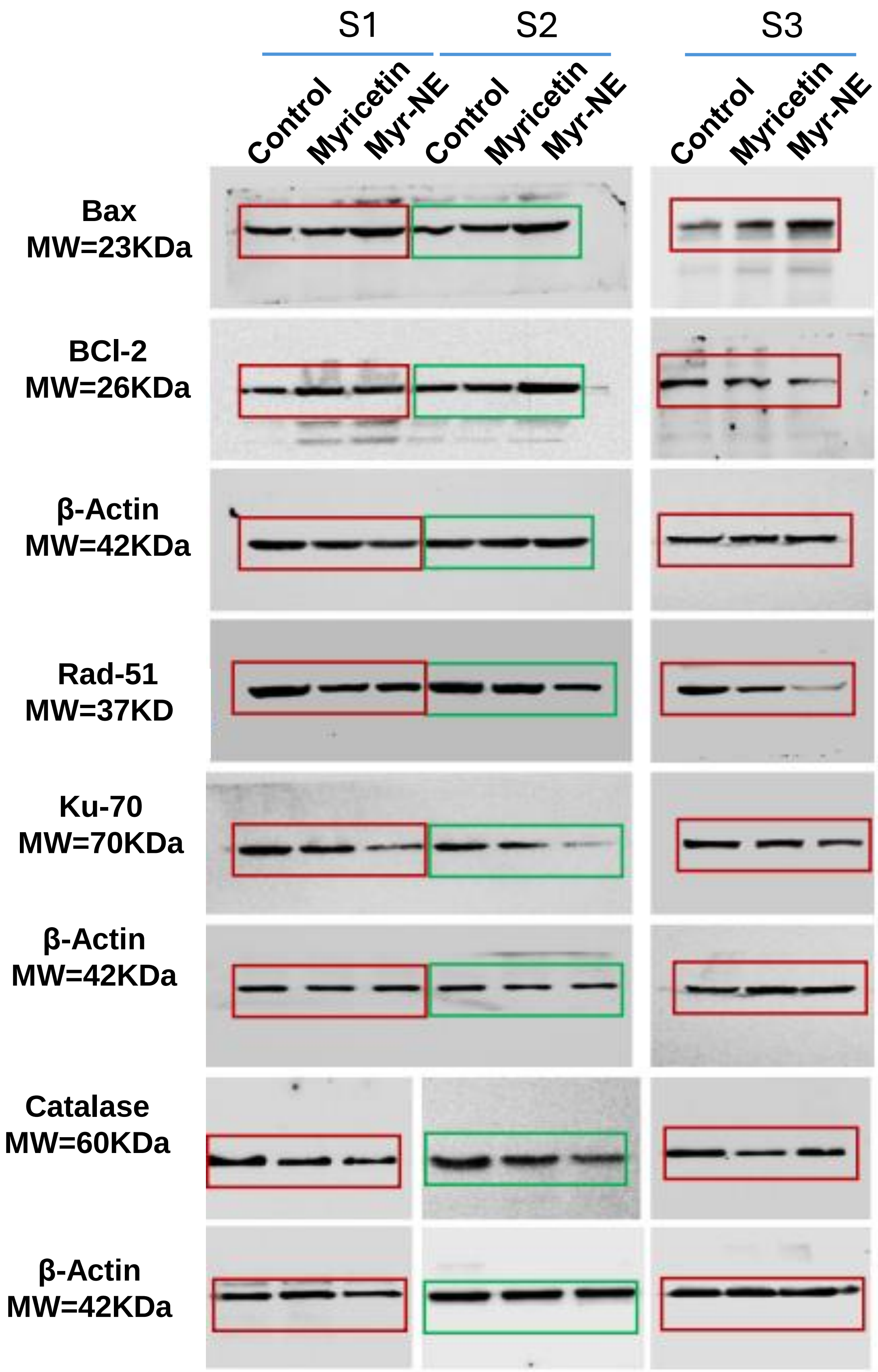


**Supplementary Figure 2.** Three blot panels of indicated proteins presented with three independent sets of tumor samples obtained from Control, Myricetin, and Myr-NE tumor xenografts (n=3). Green outline blot images of all the indicated proteins are presented in the main **figure (Fig.5 B, D. & F)** of the manuscript.

# Supplementary Figure 3

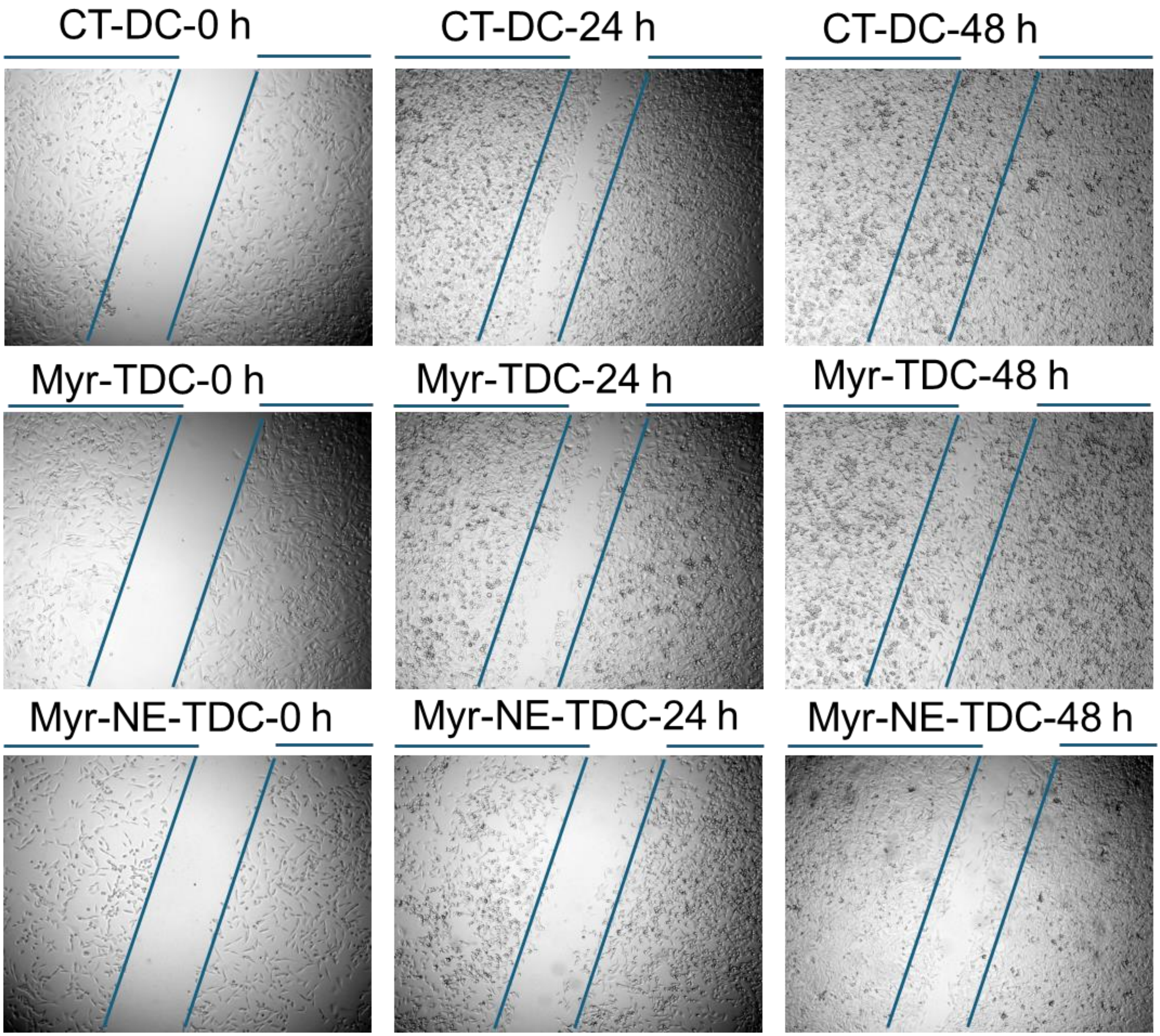


**Supplementary Figure 3:** Bright-field microscopy images of the scratch assay were captured at 10× 10 X magnification. These images were cropped and incorporated into the main manuscript as **Figure 4A.**

# Supplementary Figure 4

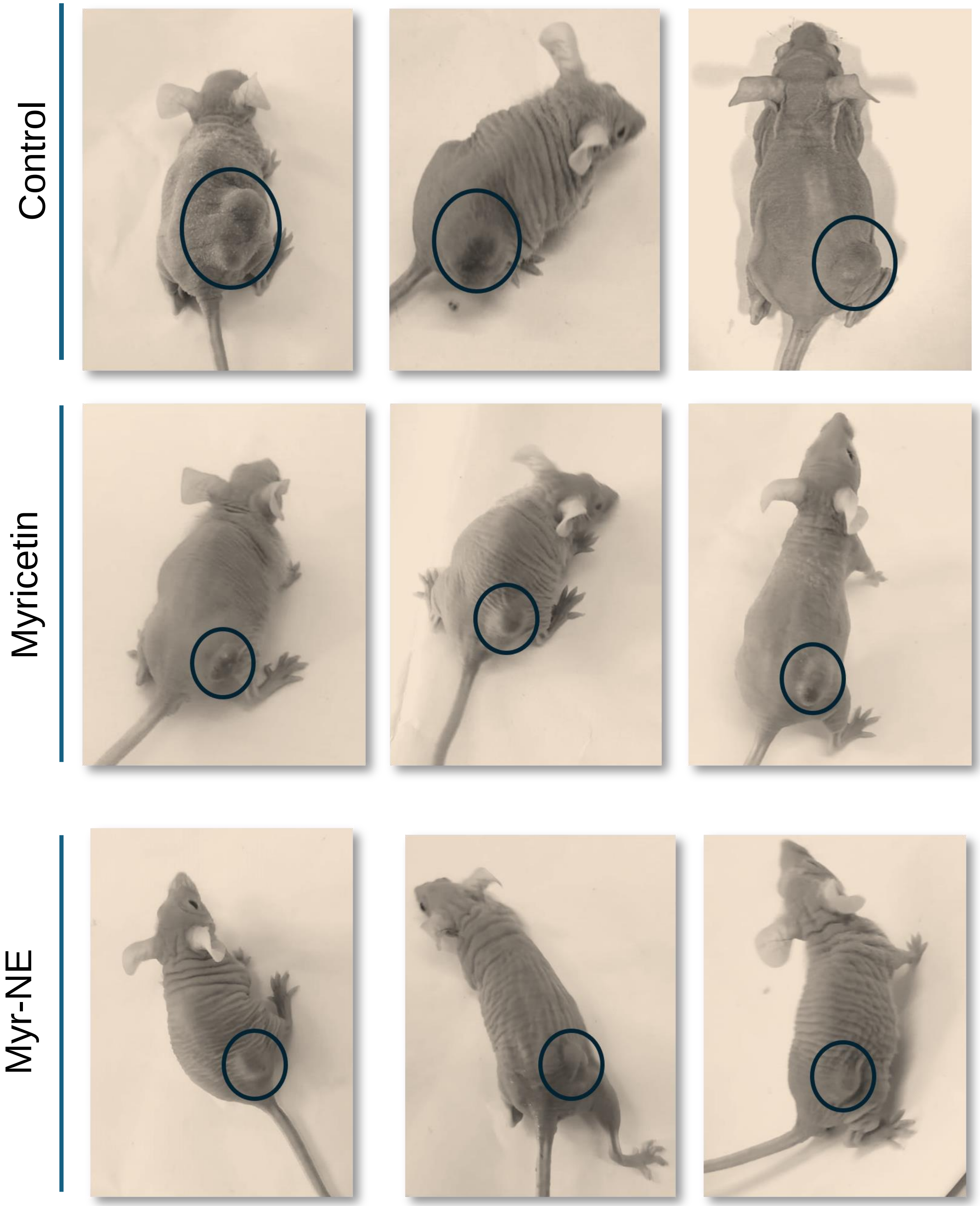


**Supplementary Figure 4:** Representative images of unanesthetized mice from each experimental group showing tumor burden and variability in tumor growth on day 21 post tumor implantation.
